# Harnessing T-Cells for Enhanced Vaccine Development against Viral Infections

**DOI:** 10.3390/vaccines12050478

**Published:** 2024-04-29

**Authors:** Zhen Zhuang, Jianfen Zhuo, Yaochang Yuan, Zhao Chen, Shengnan Zhang, Airu Zhu, Jingxian Zhao, Jincun Zhao

**Affiliations:** 1 State Key Laboratory of Respiratory Disease, National Clinical Research Center for Respiratory Disease, Guangzhou Institute of Respiratory Health, the First Affiliated Hospital of Guangzhou Medical University, Guangzhou 510182, China; zhuangzhen@gzhmu.edu.cn (Z.Z.); zhuo_jianfen@gzlab.ac.cn (J.Z.); yuanyc13@gzhmu.edu.cn (Y.Y.); 2021310831@stu.gzhmu.edu.cn (Z.C.); zhangshengnan@gzhmu.edu.cn (S.Z.); zhu_airu@gzlab.ac.cn (A.Z.); zhaojingxian@gird.cn (J.Z.); 2 Guangzhou National Laboratory, Guangzhou 510005, China

**Keywords:** vaccine, T-cell epitope, T-cell-based vaccine, viral infection

## Abstract

Despite significant strides in vaccine research and the availability of vaccines for many infectious diseases, the threat posed by both known and emerging infectious diseases persists. Moreover, breakthrough infections following vaccination remain a concern. Therefore, the development of novel vaccines is imperative. These vaccines must exhibit robust protective efficacy, broad-spectrum coverage, and long-lasting immunity. One promising avenue in vaccine development lies in leveraging T-cells, which play a crucial role in adaptive immunity and regulate immune responses during viral infections. T-cell recognition can target highly variable or conserved viral proteins, and memory T-cells offer the potential for durable immunity. Consequently, T-cell-based vaccines hold promise for advancing vaccine development efforts. This review delves into the latest research advancements in T-cell-based vaccines across various platforms and discusses the associated challenges.

## 1. Introduction

Vaccines, biological agents employed in the prevention of infectious diseases, function by stimulating the host immune system to generate immune responses against specific pathogens. They play a crucial role in bolstering resistance, reducing disease incidence, and mitigating transmission risks, thereby safeguarding global public health [1]. Their origins can be traced back to the late 18th century, credited to the pioneering work of British physician Edward Jenner. In 1796, Jenner successfully employed the cowpox virus as a vaccine to prevent human smallpox infection, marking the genesis of modern vaccinology [2].

Vaccine-induced responses typically involve B-cell-mediated antibody responses and T-cell responses. While a robust antibody response has historically been a major focus in vaccine development, recent years have seen increased attention on the protective role mediated by T-cell responses. This has led to a growing focus on the development of T-cell-based vaccines [2]. The aim is to develop vaccines capable of inducing sufficient quantities of specific CD4^+^ and/or CD8^+^ T-cells with the necessary phenotype and effector functions. These induced specific T-cells can directly promote pathogen clearance through cell-mediated effector mechanisms [3], emphasizing beyond the helper role of CD4^+^ T-cells for B-cells. The protective potential of T-cell-based vaccines has been demonstrated in the real world, notably with the tuberculosis vaccine, Bacille Calmette-Guérin (BCG). First used in the 1920s and still in use today, BCG is considered one of the first vaccines to confer protection primarily through inducing T-cell responses [2], despite this not being its original development goal. Mycobacterium tuberculosis, being an intracellular pathogen, cannot be recognized by antibodies and is only controlled through T-cell-mediated effector mechanisms [4,5].

Cellular immunity constitutes a vital component of the host’s antiviral defense mechanism, involving CD4^+^ and CD8^+^ T-cells. Multiple studies underscore the pivotal role of virus-specific T-cells in orchestrating immune protection and regulation against viral infections. CD8^+^ T-cells, also known as cytotoxic T-lymphocytes (CTLs), are indispensable for combatting viral infections. In addition to their cytotoxic function, they secrete cytokines like TNF-α and IFN-γ, endowed with antiviral properties, thereby aiding in the elimination of viruses [6]. CD4^+^ T-cells also play a crucial role in combatting virus invasion. Functioning as T-helper cells (T_H_), they furnish costimulatory signals via CD40/CD40L signaling to antigen-presenting cells (APCs) during B-cell and CD8^+^ T-cell priming [7,8]. Notably, in mice, some CD4^+^ T-cells exhibit cytotoxic capabilities by releasing perforin, providing direct defense against influenza A virus (IAV) infection [9]. Studies have revealed that pre-existing CD4^+^ T-cells in humans respond to pandemic 2009 H1N1 peptides, correlating with reduced virus shedding and milder illness during IAV infection [10]. Additionally, research confirms the existence of protective cross-reactive T-cell responses among human coronaviruses [11,12,13], mirroring the diverse protective effects of cross-reactive T-cells against the influenza virus (IV) [14,15]. Notably, specific memory T-cell populations boast an extended lifespan, with highly conserved T-cell epitopes, rendering viruses more prone to evading humoral immunity over T-cell immunity [16,17,18,19,20,21]. Given the advantages of T-cell responses, the field of vaccine research is increasingly gravitating towards eliciting T-cell responses. Consequently, what attributes characterize T-cell-based vaccines? Subsequent sections will delve into the distinctive features of T-cell-based vaccines.

## 2. Characteristics of T-Cell-Based Vaccines

1. Neutralizing antibodies typically recognize spatial epitopes located in specific regions of viral surface proteins that are involved in the initial interaction with host cells [22,23]. A single amino acid mutation may lead to the evasion of neutralizing antibody recognition. Pre-existing antibodies in the host may induce antigenic drift, as notably observed in IAV and coronaviruses [24,25]. In contrast, the epitopes recognized by T-cells are mostly linear epitopes that can be distributed on the surface and inside viral particles, expressed in infected cells, and presented by antigen-presenting cells (APCs). Point mutations outside the anchored residues can also be recognized by T-cells; therefore, the T-cell antigen repertoire is broader and less susceptible to antigenic drift [25]. Consequently, T-cell-based vaccines are expected to confer protection against infections caused by rapidly mutating viruses.
2. Viruses within the same genus typically harbor conserved proteins [26], which can be strategically harnessed in T-cell-based vaccines to elicit a broad-spectrum cross-reactive T-cell response effective against various viruses within the genus, as T-cells recognize antigen motifs. These highly preserved proteins are commonly found within virus particles and are distinct from the proteins that can be targeted by neutralizing antibodies during the viral invasion process [22]. For instance, the N protein of coronaviruses is typically conserved [27], yet neutralizing antibodies directed against it are rare.
3. Infections involving intracellular pathogens pose a challenge for antibody-mediated clearance as antibodies cannot access the pathogens within infected cells. In such cases, the cytotoxic activity of specific T-cells becomes pivotal. For instance, in HIV infection, HIV-specific CD8^+^ T-cell-mediated cytotoxic immunity can prevent disease progression and virus transmission, presenting opportunities for therapeutic and prophylactic antiviral interventions [28,29,30].
4. Virus-specific memory T-cell responses have the potential to confer prolonged protection. Studies with 6-year and 17-year follow-ups of SARS survivors revealed long-lasting specific T-cell responses, even after antibody levels have waned to undetectable levels [31,32].
5. Through immune modulation, tissue-resident memory (TRM) T-cells can be induced to provide enduring immunosurveillance and protective functions at local tissue sites, crucial in combating viruses invading local tissues [33]. Studies on various viruses, such as herpes simplex virus [34], vaccinia virus [35], and some respiratory tract infections [36,37], have demonstrated the potent protective role of TRM T-cells at tissue sites.
6. However, it is important to note that T-cell-based vaccines also face limitations. Historical success in preventing diverse infections has been limited, and while research on T-cell-based vaccines in the HIV field has been ongoing, clinical application remains challenging [28]. Factors like HLA restrictions constrain the applicability of T-cell-based vaccines [28], and T-cell-mediated immunity lacks the capacity to neutralize cell-free virus particles.

**Figure 1:**
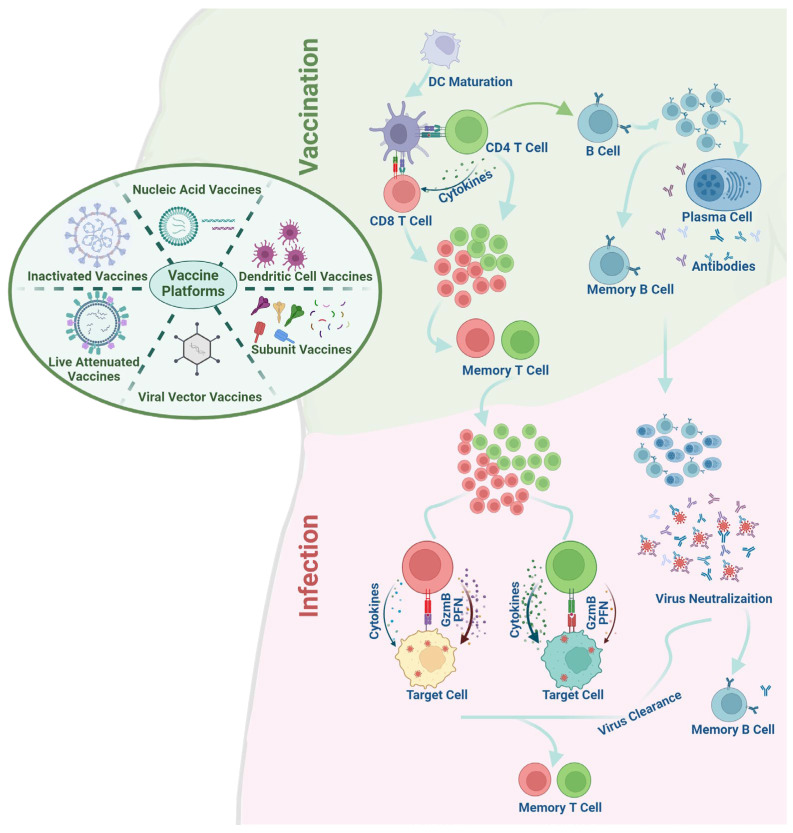
Diagram of Vaccine-Induced Immune Response. This diagram provides an overview of various vaccine platforms and illustrates the host immune responses they elicit, emphasizing adaptive immune responses. Vaccination: In summary, dendritic cells (DCs) capture and present peptides to CD4^+^ and CD8^+^ T-cells via surface MHC II and MHC I molecules, thereby initiating the activation of naive CD4^+^ and CD8^+^ T-cells. Following activation, T-cells undergo proliferation and differentiate into effector T-cells. While the majority of effector T-cells undergo apoptosis, a small fraction differentiates into memory T-cells. With antigen stimulation and the assistance of CD4^+^ T-cells, B-cells become activated, proliferate, and further differentiate into plasma cells and memory B-cells. Infection post-vaccination: Pre-existing antibodies neutralize the viruses upon infection. Memory B-cells are rapidly activated and differentiate into plasma cells, which produce a substantial quantity of antibodies, effectively eliminating pathogens through neutralization or alternative mechanisms. Concurrently, pre-existing memory CD4^+^ and CD8^+^ T-cells are promptly recalled, reactivated, proliferated, and differentiated into effector cells. These effector cells execute their functions by releasing cytokines and/or cytotoxic molecules. Following virus clearance, a fraction of effector T-cells undergo further differentiation into memory T-cells, which potentially provide extended protection. GzmB: Granzyme B; PFN: Perforin.

Given the current lesser prevalence of T-cell-based vaccines compared to antibody vaccines, comprehensive details on T-cell-based vaccine platforms remain scarce. This review endeavors to furnish an encompassing view of T-cell responses elicited by diverse vaccine platforms. Figure 1 provides an overview of the host immune responses in vaccination and infection post-vaccination, emphasizing adaptive immune responses. Through this overview, we aim to assess the ongoing progress of these platforms, evaluate their respective strengths and limitations in development, and discuss the hurdles encountered in the pursuit of T-cell-based vaccine development.

## 3. Research Progresses on T-Cell Response Induced by Different Types of Vaccines

### 3.1. Inactivated Vaccines

Inactivated vaccines are formulated by subjecting the live virus cultures to a sequence of purification technologies, subsequently rendering them non-infectious through physical and chemical means. These vaccines are characterized by their composition, which closely resembles the natural virus structure. With a lengthy history of licensing and established safety profiles, inactivated vaccines for viral diseases have garnered trust. While they predominantly elicit a humoral immune response, certain vaccines, such as inactivated IV and SARS-CoV-2 vaccines, have demonstrated the ability to induce T-cell responses as well.

Influenza virus vaccines (IVVs) currently in use come in two forms: inactivated or live attenuated, targeting tri- and tetravalent vaccines of IV A and B [38]. Inactivated IVVs incorporate epitopes derived from viral surface proteins hemagglutinin (HA) and neuraminidase (NA), stimulating the production of antibodies that target HA proteins crucial for combating viral infections [39]. While existing inactivated IVVs primarily induce CD4^+^ T-cell responses to HA and nucleoprotein (NP) [40,41], CD8^+^ T-cell responses remain relatively ineffective [42,43,44]. Inactivated SARS-CoV-2 vaccines have played a crucial role in combatting the COVID-19 pandemic. In China, the widespread administration of inactivated SARS-CoV-2 vaccines from companies like Sinovac Biotech and China National Biotec Group Company Limited has demonstrated safety and elicited antibody responses in clinical trials [45,46]. Following inoculation with inactivated vaccines, the population of helper T-cells (Th1) tends to increase [47], responding by secreting interferon-γ (IFN-γ) [48,49].

In addition to inactivated IV and SARS-CoV-2 vaccines, other inactivated vaccines have found clinical applications, including those for polio, Japanese encephalitis, rabies, and hepatitis A, as well as EV71 hand, foot, and mouth disease vaccines. The advantages of inactivated vaccines include safety, easy storage, low risk of contamination, insensitivity to neutralization by maternal antibodies, and the ability to generate combined or multivalent formulations. Nonetheless, drawbacks exist, such as the requirement for large vaccination doses, a short immunization period, and a single immune pathway that predominantly stimulates humoral immunity rather than mucosal immunity.

### 3.2. Live Attenuated Vaccines

Live attenuated vaccines utilize weakened strains of viruses to trigger immune responses, constituting a common vaccination approach. Here, we discuss several attenuated live vaccines utilized for preventing viral infections, with a focus on their capacity to induce T-cell responses.

Live attenuated IVVs emulate natural IV infections, prompting the production of protective antibodies and cellular immunity. In contrast to inactivated vaccines, live attenuated vaccines elicit superior CD4^+^ and CD8^+^ T-cell responses in children, thereby enhancing cross-protection [43,50,51]. Moreover, attenuated live vaccines can sustain specific T-cell responses for six months to one year following child vaccination [52]. Zostavax, a live attenuated vaccine containing the varicella-zoster virus (VZV), is recommended for adults over 50 to prevent shingles. Immunization with Zostavax augments the multifunctional memory of CD4^+^ T-cells, broadening the T-cell receptor repertoire of antigen-specific CD4^+^ T-cells in adults. In the general population, T-cell responses peak eight days after immunization. Older individuals vaccinated with the attenuated varicella-zoster virus exhibit defects in T-memory-cell differentiation, experiencing more rapid antigen-specific T-cell loss, thus leading to incomplete protection [53,54,55]. In addition, several effective live attenuated vaccines have been developed to prevent flavivirus infections [56]. The 17D vaccine is a live attenuated vaccine developed against yellow fever virus (YFV), and three strains of 17D vaccines are presently in production [57]. The humoral and cellular immunity triggered by the 17D vaccine has been thoroughly studied in humans. In addition to the production of neutralizing antibodies, the 17D vaccine also initiates a robust, long-lasting, and multifunctional T-cell immune response [58].

Live attenuated vaccines harbor weakened or closely related viruses, such as those used in vaccines for measles, mumps, rubella, chickenpox, and shingles. This technology facilitates mass production akin to inactivated vaccines. Although live attenuated vaccines can elicit both humoral and cellular immune responses, providing prolonged protection, their storage and transportation must adhere to precise requirements due to their active nature [59]. Individuals with compromised immune systems may have a higher risk of the attenuated strain reverting to a more virulent form, which could lead to illness in vaccinated individuals. It is also important to note that even in individuals with normal immune function, disease may occur if the vaccine strain reverts to a more virulent state.

### 3.3. Viral Vector Vaccines

Viral vector vaccines show great promise as they utilize replication-deficient vectors capable of expressing foreign antigens. These vaccines can trigger both humoral and cellular immune responses without the need for additional adjuvants. These vaccines comprise viral particles that have undergone modification to include genes encoding desired antigens [60,61]. Notable viral vectors utilized in vaccine development include adenoviruses (AdVs), modified vaccinia Ankara (MVA), IV, measles virus (MeV), and lentiviruses (LVs).

AdVs are non-enveloped double-stranded DNA (dsDNA) viruses with broad host origins and multiple serotypes [62]. Initially utilized in gene therapy, AdVs have transitioned into a vehicle for vaccine delivery. Among human-infecting adenovirus types, adenovirus type 5 (Ad5) is the most prevalent. CanSino Biologics developed a single-dose vaccine employing a highly immunogenic recombinant Ad5 vector expressing the S protein of severe acute respiratory syndrome coronavirus 2 (SARS-CoV-2), which induces significant T-cell responses [63]. However, the vaccine’s immunogenicity is compromised due to the potential pre-existing immunity in a large population from prior Ad5 exposure. Conversely, Ad26 is less common than Ad5 [64]. Janssen’s Ad26.COV2.S vector elicited a durable immune response by carrying the gene encoding the S protein of SARS-CoV-2 (SARS-CoV-2 S), resulting in humoral immunity and Th1-skewed cellular responses after a single dose [65]. To address pre-existing immunity, AstraZeneca and Oxford University employed a chimpanzee adenovirus (AZD1222) to deliver the SARS-CoV-2 S gene. A single dose of AZD1222 effectively triggered a T-cell response, with a more robust antibody response observed after the second dose [66,67]. Furthermore, intranasal administration of a chimpanzee adenoviral-vectored COVID-19 vaccine (ChAd-SARS-CoV-2-BA.5-S) intranasally prompted a strong mucosal antibody response and cross-reactive memory T-cell responses [68]. Protection notably declined upon memory CD8^+^ T-cell depletion before XBB.1.5 infection [68]. Beyond COVID-19 vaccines, AdV vectors have been applied for Ebola virus (EBoV) [69,70,71], Angola Marburg virus (MARV) [72], Rift Valley fever virus (RVFV) [73], Lassa virus (LASV) [74], and Crimean-Congo hemorrhagic fever virus (CCHFV) [75] vaccine development.

MVA serves as a robust foundational vector for vaccines, owing to its stability, immunogenicity, high safety profiles, and notably, genome-coding capabilities [76]. Preclinical studies in mice and macaques have demonstrated that immunization with an MVA-delivered stabilized SARS-CoV-2 S, alone or in combination with the N protein, elicited robust CD8^+^ T-cell responses [77,78]. Moreover, an MVA-based HIV vaccine candidate has exhibited remarkable safety in clinical trials involving 500 participants, including HIV patients and immunocompromised individuals [79,80]. Similarly, other poxvirus-based vaccines, such as the temperature-stable replicating vaccinia virus vector expressing the SARS-CoV-2 receptor-binding domain (RBD), have shown the ability to induce T-cell responses without compromising safety [81]. Overall, these vaccinations hold significant potential to emerge as highly effective shields against diseases caused by poxvirus and other pathogen infections.

T-cell-based vaccinations against pathogen infections can also utilize other viral vectors, including the IV and the measles virus (MeV). In the fight against COVID-19, IFV-based vaccines have shown effectiveness both independently and as booster vaccines. One such example is scPR8-RBD-M2, a single-round replication IFV-based COVID-19 vaccine. In mice, with two doses of intranasal (i.n.) immunization with this vaccine induced cellular, humoral, and mucosal immune responses [82]. Additionally, a live attenuated IV vector-based SARS-CoV-2 RBD vaccine elicited mucosal RBD-specific IgA and IgG responses, as well as specific T-cell responses in hamster lungs [83]. Similarly, a COVID-19 vaccine delivered by live MeV vectors expressing the prefusion-stabilized S protein demonstrated potential in mice by inducing Th1-biased T-cell responses [84,85]. Live attenuated MeV-based vaccines have been among the most effective and safe human vaccines in clinical use [86]. MeV-based vaccines targeting MERS-CoV [87], SARS-CoV [88], West Nile virus [89], and Chikungunya virus [90] have been developed, inducing robust levels of neutralizing antibodies (NAbs) and T-cells. Highly immunogenic and efficacious MeV-based vaccine candidates may be incorporated into regular MMR (measles, mumps, rubella) vaccination regimens, providing additional defense against various illnesses.

LVs, initially derived from HIV, are also ideal platforms for vaccines due to their robust immunogenicity and capacity to elicit immunological responses even after a single dose of immunization. LV-based vaccines have made significant strides in pre-clinical studies targeting SARS-CoV-2 and Zika viruses [91,92,93]. An intranasal booster utilizing LV-based vaccinations containing the S of the Beta variant has bolstered systemic and lung-resident T- and B-cell immunity, offering protection against Omicron variant infection [94]. However, concerns have been raised regarding the integration of LV vector-based vaccines into the host genome. To address this, a non-integrative LV T-cell antigen-based vaccine for human coronaviruses has been developed, generating protective T-cell immunity and providing a broader defense against SARS-CoV-2 variants [95]. Furthermore, a non-integrating LV-based Zika vaccine encoding the pre-membrane and envelope glycoproteins of Zika virus strains elicited robust neutralizing antibody titers and conferred full protection against the Zika challenge [92]. These findings suggest promising prospects for the development of vaccines utilizing LVs.

In general, viral vector vaccines are considered safe and effective in activating both innate and adaptive immunity. Their capacity to replicate natural infection processes, effectively deliver antigens, and prompt robust immune responses renders them valuable tools in vaccine development. However, challenges such as pre-existing immunity and limitations in vector packaging must be overcome to enable broader utilization of viral vectors in vaccine design. Furthermore, thorough studies on the long-term safety and efficacy are still needed.

### 3.4. Subunit Vaccines

Non-viral vaccines are a type of vaccine that solely utilize specific components (subunits) of viruses or bacteria essential for immune system recognition. Unlike traditional vaccines, they do not contain the entire microorganism or utilize benign viruses as vectors. This section will explore various subunit vaccines and delve into the advancements in peptide vaccine research.

Virus-like particle (VLP) vaccines are a type of subunit vaccine that mimics virus morphology without containing a viral genome. They are produced by expressing antigen proteins in eukaryotic or prokaryotic systems, allowing for the formation of particles with self-assembling antigen proteins [96]. Dendritic cells take up VLPs, process them, and present them on MHC I and II to trigger the CD8^+^ and CD4^+^ T-cell responses [97,98,99,100]. Many VLP vaccines, including those targeting hepatitis B (HBV), human papillomavirus (HPV), IV, Zika virus, etc., are now in clinical use or undergoing clinical trials.

Nanoparticle (NP) vaccines are created by chemically cross-linking proteins and carrier molecules to boost immune response and prevent antigen breakdown. In certain studies, researchers have incorporated immune receptor agonists (ligands of Toll-like receptor) into NP vaccines to increase dendritic cell activation and stimulate robust CD8^+^ and CD4^+^ T-cell reactions [101,102,103].

Recombinant protein vaccines are a type of subunit vaccine that is currently extensively researched. Flublok, for instance, is a recombinant entire quadrivalent-HA protein subunit vaccine designed to prevent IV infections. It is notable for being the first licensed IVV to utilize recombinant viral proteins instead of antigens taken from live influenza viruses [104]. In comparison to split vaccines, Flublok demonstrates superior effectiveness in stimulating IV-specific CD4^+^ T-cells and CD4^+^ T-cell-dependent antibody responses in humans [105]. Another noteworthy vaccine is the gE protein-based Herpes zoster (HZ) vaccine, which incorporates a second-generation lipid adjuvant (SLA), a synthetic TLR4 ligand, within an oil-in-water emulsion (SLA-SE). This formulation induces polyfunctional CD4+ T-cell responses in both young and aged mice, with sustained T-cell responses observed up to 5 months post-immunization [106]. Likewise, other VZV recombinant protein vaccines have the capability to provoke multifunctional and enduring T-cell responses [107,108].

While VLPs, NPs, and recombinant protein vaccines can trigger T-cell immune responses, their antigen designs primarily target inducing humoral immunity. A novel type of vaccine, known as peptide vaccine, focuses on epitope peptide design rather than protein design. Peptide vaccines utilize peptides, typically derived from pathogen proteins and shorter than full proteins, to elicit an immune response against infections. They show potential in developing vaccines for infectious diseases, with numerous reports highlighting their effectiveness. T-cell epitope-targeting vaccines, such as CoVac-1, incorporate multiple T-cell epitopes from various SARS-CoV-2 viral proteins along with toll-like receptor 1/2 agonist XS15. CoVac-1 can stimulate robust SARS-CoV-2 T-cell immunity and potentially offer cross-reactivity to SARS-CoV-2 variants of concern due to the conservation of selected T-cell epitopes [109]. In patients with B-cell/antibody deficiency, a single dose of CoVac-1 can induce extensive and efficient T-cell responses with a favorable safety profile [110]. Another example is Multimeric-001, a synthetic recombinant peptide vaccine comprising nine T-cell and B-cell epitopes sourced from IV NP, M1, and HA proteins. When paired with the adjuvant Montanide ISA 51VG, Multimeric-001 has demonstrated the capacity to induce cellular responses in both healthy and older individuals [111,112].

Compared to whole-virus vaccines, non-viral vaccines are considered safer and more stable. They provide long-lasting immunity with a single dose, eliminating the need for repeated booster shots. Subunit vaccines can be manufactured on a large scale using vectors such as *E. coli*, rod-shaped viruses, yeast, and others. These vaccines not only elicit humoral immune responses but also activate T-cell-mediated immunity.

### 3.5. Dendritic Cell Vaccines

Dendritic cells (DCs) are tissue-resident and circulating cells that sense microbes, initiate innate immune defense reactions, and present microbial proteins to T-cells to initiate adaptive immune responses [113]. DCs are the most effective antigen-presenting cells capable of inducing CD4^+^ and CD8^+^ T-cell responses. Due to their unique characteristics, DC vaccines have been developed for treating cancer and infectious diseases. In clinical immunotherapies and the regulation of the anti-tumor immune response, monocytes are isolated from patient peripheral blood mononuclear cells (PBMCs) and differentiated into DCs through incubation with granulocyte-macrophage colony-stimulating factor (GM-CSF) and other cytokines for about 5 days. Subsequently, DCs are loaded with inactivated autologous viruses, transfected with antigen-encoding RNA or DNA, or pulsed with viral antigens [114]. Recently, DC vaccines have been developed for various viruses, including HIV, HCV, SARS-CoV-2, HBV, IV, and LCMV.

In chronic viral infection diseases, DC vaccines are employed for the prevention and treatment of HIV and HCV. In HIV prevention and treatment, research has demonstrated that an autologous DC HIV-1 vaccine, loaded with either autologous HIV-1–infected apoptotic cells or inactivated HIV-1, can induce polyfunctional HIV-1 specific CD4^+^ T-cell and gag–specific CD8^+^ effector T-cell responses [115]. Furthermore, DCs loaded with recombinant proteins have been shown to elicit HIV-specific lymphocyte proliferation responses, resulting in enhanced production of IL-2, TNF-α, and IFN-γ [116]. Additionally, DCs have the capability to present viral peptides as immunogens, including peptide pools [117], single epitopes [118], and HIV-1-antigen lipopeptides [119]. Notably, all peptide-presenting DCs were capable of stimulating HIV-1-antigen-specifific T-cell responses. Currently, DCs have been effectively utilized to express HCV structural proteins, such as HCV-core protein and NS3 protein, using various systems like lentiviral systems [120], recombinant adenoviral vectors [121], adenovirus systems [122], and recombinant adeno-associated virus systems [123]. This approach has shown significant success in generating antigen-specific CD4^+^ and CD8^+^ T-cell responses [124]. Additionally, loading DCs with HCV antigens has been demonstrated to elicit robust humoral and cellular immune responses in mice [125,126]. In a phase I clinical trial involving six HLA-A2 patients, DCs pulsed with lipopeptides containing a CD4^+^ T-cell epitope and HLA-A2-restricted CD8^+^ T-cell epitope, along with the lipid Pam2Cys, were capable of inducing specific CD8^+^ T-cell responses [127,128].

DC vaccines are also used in acute viral infections, including SARS-CoV-2 and IV. Several phase I-II trials (NCT04386252, NCT05007496, NCT04690387, NCT05007496.) have been conducted to evaluate the efficacy of DC vaccines in preventing COVID-19. Data from trials (NCT04386252, NCT04690387, and NCT05007496) demonstrated increased levels of anti-RBD antibodies [129], while data from trial NCT05007496 show that subjects exhibited reactivity to the SARS-CoV-2 S protein. In another phase I/II trial (NCT04276896), the LV-SMENP-DC vaccine was developed by modifying DCs using LV expressing the SARS-CoV-2 minigene SMENP and immune modulatory genes. The LV-SMENP-DC vaccine not only triggers the production of neutralizing antibodies but also elicits specific CD8^+^ T-cell responses [130,131]. China Celartics Biopharma is focusing on utilizing engineered DCs with NP to induce NP-specific CD8^+^ T-cells [132]. IV DC vaccines have the potential to induce HA-specific antibodies and T-cell responses [129]. Lentiviral vector-transduced DCs with lymphocytic choriomeningitis virus (LCMV) GP33–41 peptides can elicit a protective response to LCMV infection by enhancing the CD8^+^ T-cell responses [133].

The DC vaccine offers the advantage of individualized preparation tailored to the patient’s specific condition, providing long-lasting immune effects and immune specificity, particularly in T-cell responses. However, in clinical practice, the conventional DC manufacturing method necessitates a significant amount of blood, proving cost-prohibitive, time-consuming, and potentially unable to fully replicate the essential properties of naturally occurring dendritic cells. Additionally, the production of monocyte-derived dendritic cells may pose limitations in addressing highly pathogenic viruses, requiring specialized PC3 facilities and involving labor-intensive and complex operations, thereby restricting the application of DC as a preventive vaccine against viral infections.

### 3.6. Nucleic Acid Vaccines

Nucleic acid vaccines, represent a promising strategy in the battle against viral infections, particularly COVID-19. Nucleic acid vaccines fall into two main categories: DNA vaccines and RNA vaccines. RNA vaccines are further categorized into linear mRNA, circular RNA (circRNA), and self-amplifying mRNA (saRNA). These vaccines induce T- and B-cell responses by introducing foreign genes into recipients, prompting the production of antigenic proteins.

DNA vaccines utilize DNA plasmids as vectors to deliver immunogenic antigens, which are encoded in genes and must be electroporated into cells for effective delivery. This approach induces both humoral and cell-mediated immune responses [134,135]. Research on DNA-based COVID-19 vaccines has shown promising results in triggering T-cell-mediated immunity. DNA vaccines like COVID-eVax and GX-19N have demonstrated efficacy in eliciting SARS-CoV-2-specific T-cell responses [136,137]. DNA vaccines have also been successfully developed against SARS-CoV, with results indicating robust humoral and cellular immune responses in mice, macaques, and camels [138,139]. Additionally, the first DNA vaccine candidate against MERS-CoV, GLS-5300, entered clinical trials and was well tolerated without any major side effects [140,141]. Despite some safety concerns, DNA-based vaccines containing T-cell epitopes are showing great promise. Additionally, DNA vaccines present several advantages compared to traditional and mRNA vaccines, including rapid manufacturing, cost-effectiveness, and enhanced stability for transportation and storage. Nonetheless, a significant challenge of DNA vaccination lies in the limited immune responses observed in humans so far [134,142]. This is exacerbated by the fact that the effectiveness of a DNA vaccine in priming immune responses may only become apparent following the administration of a heterologous boost, which is suboptimal in situations necessitating a prompt immune response, such as during an outbreak [143].

RNA vaccines, on the other hand, offer the advantage of rapid development and flexibility. To date, mRNA therapeutics are the most advanced application for infectious diseases. mRNA vaccines, such as the COVID-19 vaccines developed by Pfizer BioNTech and Moderna, do not require the cultivation of viruses or preparation of specific proteins, enabling them to be designed and produced more rapidly [144,145,146]. mRNA vaccines have the ability to elicit strong CD4^+^ and CD8^+^ T-cell responses, providing lasting immunity against infections [147,148]. When comparing mRNA-1273, BNT162b2, Ad26.COV2.S and NVX-CoV2373, mRNA vaccines showed a higher memory CD4^+^ T-cell response and a similar memory CD8^+^ T-cell response compared to the viral vector-based immunization [149]. The mRNA technologies have also been applied in the development of vaccines for IV [150,151,152], Zika [153,154], HIV [155], Respiratory syncytial virus (RSV) [156] and EBoV [157]. In phase I clinical trials, mRNA vaccines for RSV (mRNA-1777) elicited a robust humoral response and a CD4^+^ T-cell response to RSV F peptides with no serious adverse events reported [158]. In addition, mRNA vaccines against EBOV elicited robust expression of IFN-γ and IL-2 by CD8^+^ and CD4^+^ T-cells [159]. CircRNA, unlike linear mRNA, are stable noncoding RNAs with a covalently closed ring structure that shields them from degradation. CircRNA vaccines have shown stronger and longer-lasting immunogenic responses compared to mRNA vaccines, inducing Th1-biased T-cell responses [160]. Moreover, genetically modified replicons derived from single-strand RNA viruses, known as saRNA vaccines, can prolong antigen expression and stimulate humoral and cellular immune responses [161]. For example, mice injected with the saRNA vaccine containing the stabilized SARS-CoV-2 S protein showed pronounced CD8^+^ and CD4^+^ T-cell responses even at low doses [162]. Another saRNA vaccine in mice, ZIP1642, encoding both S-RBD and N antigens, induced Th1-skewed cytokine responses along with specific T-cell responses [163]. Ongoing research in this field indicates that such vaccine approaches could be valuable in combating COVID-19 and potentially other viral infections in the future. However, RNA vaccines require storage at low temperatures and may present challenges for large-scale production and the establishment of mucosal immunity. The optimization of the nucleic acid sequence of the antigen is necessary to enhance antigen expression. Long-term safety and efficacy studies are essential in the coming years to safeguard the well-being of global populations.

Table 1 provides an overview of various vaccine platforms, describing their mechanisms of action, adjuvant requirements, induction of antibody and T-cell responses, and advantages and challenges.

## 4. Challenges in T-Cell-Based Vaccine Development

### 4.1. Identification and Selection of T-Cell Epitopes

T-cell epitopes form the foundation of T-cell-based vaccine development. Due to the MHC molecule restriction of T-cell responses, T-cell epitopes vary across different species or individuals. Thus, identifying specific T-cell epitopes is crucial for candidate virus T-cell vaccine development. Current methods for identifying T-cell epitopes include bioinformatics predictions and peptide library screenings [13,164]. While prediction methods offer speed, the predicted epitopes may not always be authoritative T-cell epitopes. Conversely, peptide library screenings are more accurate but are time-consuming and labor-intensive.

The functionality of T-cells specific to different epitopes of a virus can be either protective or pathogenic—for instance, CD8^+^ T-cells targeting PA_224–233_ of IV can impede virus clearance, whereas NP_366–374_-specific CD8^+^ T-cells can expedite IV elimination [165]. Once the T-cell epitopes are known, determining whether they are protective or pathogenic is vital for accurate selection. However, validating T-cell functionality and understanding the immunological mechanisms involved can be challenging, time-consuming, and laborious.

### 4.2. Time Constraint: “Swift Deployment, Lasting Immunity”

When discussing the timeframe for vaccine development and the duration of vaccine protection, the phrase “swift deployment, lasting immunity” succinctly encapsulates the concept. “Swift deployment” denotes the ability to rapidly develop T-cell epitope vaccines in response to viral pandemics. This involves promptly identifying epitopes, selecting those with protective properties, efficiently choosing a suitable delivery platform, and swiftly verifying their protective efficacy. Conversely, “lasting immunity” emphasizes the necessity for T-cells to persist long-term post-vaccination, providing sustained protection to the host. Achieving both rapid development and long-lasting protection with T-cell-based vaccines poses significant challenges that require innovative solutions.

### 4.3. Choosing the Delivery System

As outlined earlier, each vaccine platform presents unique strengths and weaknesses. When crafting T-cell-based vaccines, the selection or development of an efficient delivery system for expressing or delivering epitopes is paramount. Additionally, integrating adjuvants to bolster the cellular response should be carefully considered in tandem with the chosen platform.

### 4.4. Achieving Immune Response Balance

A successful T-cell-based vaccine must strike a delicate balance in eliciting immune responses. Excessive T-cell activation can trigger inflammation, underscoring the importance of modulating the immune reaction. Research has demonstrated the essential role of adaptive immune cells in eliminating SARS-CoV-2 infection, However, this process can also result in increased inflammation and associated pathology [166]. Immunization with vaccines that selectively induced CD4 T-cell responses led to severe inflammation and mortality after a challenge with a persistent strain of chronic lymphocytic choriomeningitis virus (LCMV) [167]. Balancing the T-cell responses is pivotal in harnessing protective immunity without inciting an overly aggressive reaction. Managing this equilibrium poses a significant challenge in T-cell vaccine development.

### 4.5. Strain-Specificity and Cross-Protection Considerations

It is imperative that T-cell epitopes align with the targeted pathogen, ideally allowing T-cells to recognize viruses within the same family or across other families. Conducting a comparative analysis of T-cell epitopes from diverse strains within the target virus genus aids in selecting epitopes with minimal variability. Particularly for RNA viruses prone to mutation, prioritizing T-cell epitopes located on conserved proteins helps mitigate epitope escape. Developing an optimal T-cell epitope vaccine capable of conferring protection against infection while offering cross-protective capabilities poses a significant challenge.

### 4.6. Selection of Immunization Routes

For viruses that primarily infect via mucosal routes, such as respiratory viruses, it is crucial to consider respiratory mucosal delivery methods. Vaccination through the respiratory tract may generate trained innate immunity and optimal B- and T-cell immunity that is essential in defense against infection [168,169,170,171]. The efficacy of mucosal immunization in eliciting mucosal immune responses relies on several factors, including the vaccine platform itself. For instance, mRNA vaccine platforms necessitate overcoming the substantial technological hurdle of mucosal targeting lipid nanoparticle (LNP) delivery systems.

## 5. Conclusions

T-cells are pivotal in orchestrating regulatory and protective functions within the host’s antiviral immune responses. The integration of T-cell epitopes into vaccine development for infectious diseases signifies a progressive shift in vaccine design. Nevertheless, the development of T-cell-based vaccines or those integrating T-cell epitopes faces several challenges. Despite these obstacles, it is expected that scientists will progressively overcome these hurdles, ultimately paving the way for the creation of potent, enduring, and broad-spectrum T-cell-based vaccines.

## Figures and Tables

**Table 1:** Detailed overview of diverse vaccine platforms, including their mechanisms of action, requirement for adjuvants, stimulation of antibody, and T-cell responses, as well as their benefits and obstacles.

Vaccine Platforms	Mechanism of Action	Need for Adjuvants	Induction of Humoral Responses	Induction of T-Cell Responses	Advantages	Challenges
Inactivated Vaccines	Antigenic substances composed of inactivated material from a pathogen	Yes	Yes	Yes	Safety, convenient storage, minimal contamination risk, resistance to neutralization by maternal antibodies, and capacity to produce combined or multivalent formulations.	High vaccination doses, a short immunization period, and a single immune pathway that predominantly stimulates humoral immunity rather than mucosal immunity.
Live Attenuated Vaccines	A living but weakened version of the pathogen	No	Yes	Yes	A single dose provides potent, long-lasting protection.	Storage and transportation must adhere to precise requirements, higher risk reverting to a more virulent form in individuals with immunodeficiency, and possible pathogenic in individuals with normal immune function if the vaccine strain re-verts to a more virulent state.
Viral Vector Vaccines	Display viral proteins in non-replicative virus vectors	No	Yes	Yes	Safe, capacity to replicate natural infection processes, effectively deliver antigens, and prompt robust immune responses.	Pre-existing immunity and limitations in vector packaging, unknown long-term safety, and efficacy.
Subunit Vaccines	Utilize specific components (subunits) of virus	Yes	Yes (T-cell epitope vaccines do not elicit specific antibodies)	Yes	Safer, stable, long-lasting immunity with a single dose, less chances of side-effects.	Less immunogenic than live attenuated vaccines, particular antigen or epitopes should be identified.
Dendritic Cell Vaccines	DCs pulsed with indicated T-cell epitopes	No	No	Yes	Individualized preparation tailored to the patient’s specific condition, providing long-lasting immune effects and immune specificity, particularly in T-cell responses.	An individualized strategy requires a substantial amount of blood, proving cost-prohibitive, time-consuming, and potentially unable to fully replicate the essential properties of naturally occurring dendritic cells, limitations in addressing highly pathogenic viruses.
Nucleic Acid Vaccines	Deliver viral genetic material (DNA or mRNA) to instruct cells to produce antigens.	No	Yes	Yes	DNA vaccines: Stable, allowing for specific genes to be added or deleted as needed. mRNA vaccines: shorter manufacturing time, elicit potent immunity.	DNA vaccines: limited immune responses observed in humans, unclear long-term safety, and efficacy. mRNA vaccines: need storage at low temperatures, unknown long-term safety.
